# Patient's perspective in clinical practice to assess and predict disability in multiple sclerosis

**DOI:** 10.1038/s41598-022-23088-x

**Published:** 2022-10-29

**Authors:** S. Gil-Perotin, L. Bernad, S. Reddam, C. Ferrer-Pardo, S. Navarro-Quevedo, L. Solís-Tarazona

**Affiliations:** 1 Neuroimmunology Research Unit, Health Research Institute, Hospital Universitario Y Politécnico La Fe, Avda. Fernando Abril Martorell, 106, 46026 Valencia, Spain; 2 Neurology Department, Hospital Universitario Y Politécnico La Fe, Avda. Fernando Abril Martorell, 106, 46026 Valencia, Spain; 3 Department of Neurology, Dr. Peset Hospital, Valencia, Spain

**Keywords:** Neurology, Multiple sclerosis

## Abstract

The information provided by a person with multiple sclerosis (MS) may anticipate changes in the course of the disease. To explore the role of a set of standardized patient-reported outcomes (PRO) in predicting disability progression in MS an observational study was conducted in two cohorts of 30 and 86 persons with progressive MS (pwPMS) and relapsing MS (pwRMS), respectively. The associations between baseline clinical, biochemical variables and results on MS quality of life scale (MusiQol), Modified Fatigue Impact Scale (MFIS) and Beck Depression Inventory II (BDI-II) were analyzed. The progression of disability after 2 years of follow-up in pwRMS was investigated. We show that PRO differentiated pwRMS and pwPMS cohorts with lower MusiQoL and higher MFIS and BDI-II scores in the latter. Only MFIS was correlated with disability in pwRMS and high scores in the physical MFIS domain associated with worse performance in 9HPT, and a trend in T25FW and SDMT. Instead, the cognitive MFIS domain was correlated with CHI3L1 in cerebrospinal fluid, a biomarker of progression. At the end of the study, global MFIS and BDI-II were found to be independent risk factors for disability independent of relapse. Although all PRO measures explored were altered in pwPMS, baseline MFIS discriminated current and prospective disability in pwRMS, identifying patients at risk of progression.

## Introduction

Multiple sclerosis (MS) is a chronic, autoimmune, inflammatory, and neurodegenerative disease that affects the central nervous system (CNS). MS symptoms induce general disability, impacting quality of life (QoL) together with health costs^1^. The most frequent presentation of MS is as a two-stage disease, with early relapsing inflammation responsible for remitting MS (RMS) and, years later, in a percentage of patients, delayed neurodegeneration causing non relapsing progression of disability, the secondary progressive phase of MS (PMS)^2^. The diagnosis of MS is made according to clinical, radiological and, recently, biochemical criteria ^3^ and after diagnosis, early prediction of MS outcomes is a challenge because, until now, there is no strong candidate biomarker able to detect, early during disease course, those people with MS (pwMS) with subclinical neuronal damage and, therefore, more prone to develop clinical progression and functional impairment over time^4^.

Beyond clinical and paraclinical assessment, the most accessible and reliable source of information is the one given by the pwMS^5^. Patients describe its personal condition and relate the impact of therapies on her/his QoL visit by visit. Increasing evidence of the importance of incorporating the patient's perspective into medical decisions has contributed to a paradigm shift in health care and in the evaluation of health outcomes^6^. The most extended manner to retrieve this information directly by patients is in the form of patient-reported outcomes (PROs)^5^.

The aim of this study was to analyze a set of standardized PROs to characterize QoL, fatigue, and depression in different populations of MS (RMS and PMS), and to describe baseline clinical, radiological, biochemical characteristics, and the 2-year risk of relapse and/or progression of disability in a prospective cohort of persons with RMS (pwRMS).

## Results

### Patients with RMS and PMS have distinct PRO scores

**Figure 1:**
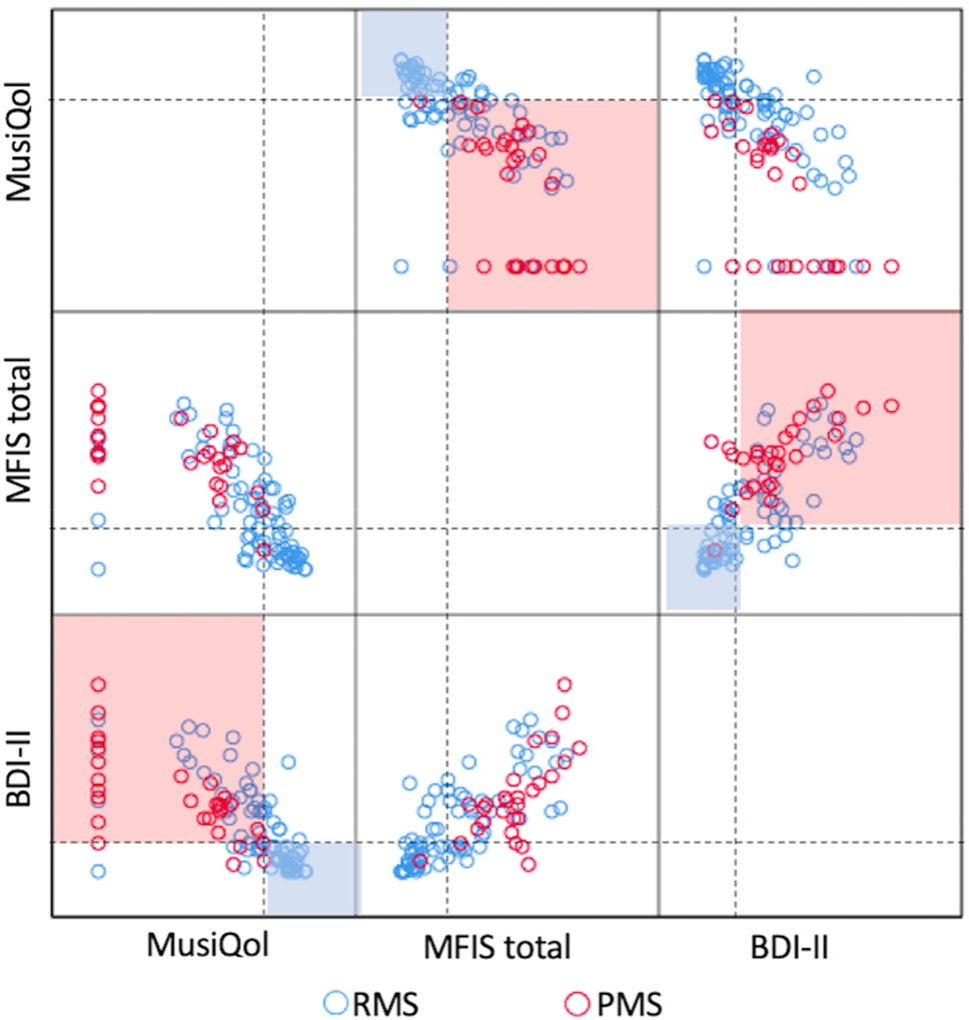
Cohort distribution based on MusiQoL, MFIS and BDI-II values and MS phenotype. A bidimensional correlation matrix shows the relationship between the PRO investigated and the distribution of pwPMS (red empty dots) and pwRMS (blue empty dots). Dashed lines represent the median values for the pwRMS cohort. Note that in each 2 × 2 graph, patients tend to distribute in two squares, the shaded in blue, were pwRMS and favorable results in the scales concentrate, and the shaded in red were pwPMS are mostly confined, corresponding to less favorable PRO scores. Interestingly, although none of the pwPMS are in the blue squares, there are several pwRMS that are included in the red squares, suggesting that these patients could be misclassified or behave differently. Abbreviations: PMS: progressive multiple sclerosis; RMS: relapsing multiple sclerosis; PRO: patient-reported outcomes; MusiQoL: Multiple Sclerosis Quality of Life; MFIS: Modified Fatigue Impact Scale; BDI-II: Beck Depression Inventory-II.

Thirty-three pwPMS and 86 pwRMS completed the PROs questionnaires MusiQol, MFIS, and BDI-II at study inclusion (*baseline*). Demographic and clinical features of both cohorts, and scores obtained from these questionnaires are listed in Tables 1 and 2, respectively. The PROs correlated with each other throughout the cohort (MusiQol vs. MFIS Rho = − 0.727 [*P* < 0.001], MusiQol vs. BDI-II Rho = − 0.738 [*P* < 0.001], and MFIS vs. BDI-II Rho = 0.777 [*P* < 0.001]), and the results recovered from the pwRMS and pwPMS groups were significantly different, with lower MusiQoL (*P* < 0.001), higher MFIS (*P* < 0.001) and higher BDI-II (*P* = 0.003) scores in pwPMS (Fig. 1).

**Table 1:** Demographic and clinical features of patients in the cohort of the study.

	PMS (N = 33)	RMS (N = 86)	*p*-value
Age at the onset of the disease, mean (SD)	34 (13.6)	26.4 (7.0)	< 0.000
Age at study inclusion, mean (SD)	56.7 (12.9)	31.7 (7.2)	< 0.000
Sex (female), number (%)	25 (48)	88 (68)	0.049
Duration of the disease (years), mean (SD)	22 (9.5)	4.9 (3.9)	< 0.000
EDSS at study inclusion, median (p25–p75)	7 (6.5–8.0)	1.5 (1–2.5)	< 0.000
Prior disease activity, number (%)	0 (0)	34 (40)	< 0.000
Disease-modifying therapies (DMT), number (%)	14 (42.4)	83 (96.5)	< 0.000
No	19 (57.6)	3 (3.5)	< 0.000
First-line DMT	2 (6)	25 (29.1)	< 0.000
High-efficacy DMT	12 (36.4)	58 (67.4)	< 0.000

N, number of patients; PMS, progressive multiple sclerosis; RMS, relapsing multiple sclerosis; EDSS, expanded disability status scale; DMT, Disease-modifying therapies; NA, not-available.

Significant values are in bold.

**Table 2:** Statistical differences between PRO scores from the pwPMS and pwRMS groups.

	PMS		RMS		*p*-value
	Mean (SD)	Median (p25–p75)	Mean (SD)	Median (p25–p75)	
MusiQol	35.5 (30.6)	51.1 (0–59.7)	67.7 (28.6)	78.1 (59.3–88.5)	< 0.000
Global MFIS	53.6 (16.4)	53 (40.5–62.5)	26.3 (22.7)	22 (6.8–38.3)	< 0.000
physical	28.9 (5.6)	29 (24.5–32)	11.7 (10.4)	9 (2–21)	0.006
cognition	18.7 (11.3)	20 (13–25)	12.3 (11.3)	9 (3–20)	< 0.000
psychosocial	6.07 (2.2)	6 (5–8)	2.3 (2.4)	2 (2–4)	< 0.000
BDI-II	21 (12.3)	19 (12.5–26.8)	13.5 (12.7)	8 (3–21)	0.003

PRO, Patient-reported outcomes; PMS, progressive multiple sclerosis; RMS, relapsing multiple sclerosis. MusiQoL, Multiple Sclerosis Quality Of Life; MFIS, Modified Fatigue Impact Scale; BDI-II, Beck Depression Inventory-II.

The *p*-value was considered significant when < 0.05.

Although, overall, pwRMS concentrated in the region corresponding to better results in QoL, lower fatigue, and mild or absent depression, the percentage of pwRMS that were beyond median values was elevated. Therefore, we investigated the relationship between baseline PRO and contemporary clinical, radiological, and biomarker parameters in pwRMS, and, at the end of the 2-year duration of the study, the disability worsening (DW) and the occurrence of clinical or radiological inflammation.

### Global MFIS and MFIS domains showed the best correlation with neurological disability in pwRMS

At baseline, EDSS was correlated with global MFIS (Rho = 0.369; *P* = 0.006) and MFIS subscales, tended to correlate with MusiQoL (Rho: − 0.298; *P* = 0.06) but did not correlate with BDI-II scores (Table 3). MusiQoL correlated with CSF NFL levels (Rho = − 0.424; *P* = 0.024) and with baseline serum NFL (Rho = − 0.385; *P* = 0.024) only in patients with contemporary disease activity.

**Table 3:**
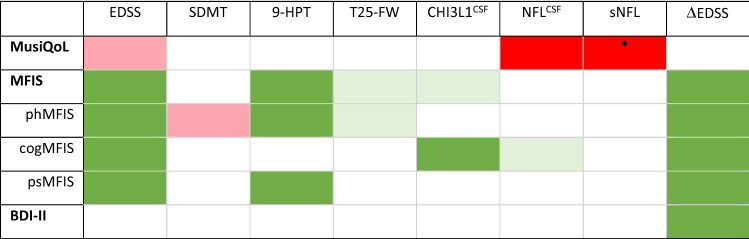
Correlation of baseline PRO with clinical variables and biomarkers.

Legend of colors: “red” shows negative correlation; “green” shows positive correlation; “light red” or “light green” shows tendency (*P* < 0.1) for negative or positive correlation, respectively. Abbreviations: MusiQoL: Multiple Sclerosis Quality of Life; MFIS: Modified Fatigue Impact Scale; phMFIS: MFIS physical; cogMFIS: MFIS cognitive; psMFIS: MFIS psychosocial; BDI-II: Beck Depression Inventory-II; EDSS: Expanded Disability Status Scale; SDMT: Symbol Digit Modality Test; 9-HPTd: 9-Hole Peg Test dominant hand; 9-HPTd: 9-Hole Peg Test non-dominant hand; T25-FW: Timed 25-Foot Walk Test; CHI3L1: chitinase3 like-1; NFL: neurofilament light chain protein; CSF: cerebrospinal fluid; sNFL: serum NFL; ∆EDSS: EDSS change.

(*)Disease activity.

Regarding other variables, global MFIS scores correlated with 9-HPT (Rho = 0.281; *P* = 0.033) and showed a trend to correlate with CSF CHI3L1 levels (Rho = 0,264; *P* = 0.096) and T25-FW (Rho = 0.214; *P* = 0.096). Physical MFIS was correlated with 9-HPT (Rho = 0.310; *P* = 0.015) with a trend for T25-FW (Rho = 0.43; *P* = 0.062) and SDMT (Rho = − 0.201; *P* = 0.076). Cognitive MFIS, instead, correlated with CSF CHI3L1 (Rho = 0.338; *P* = 0.048) with a tendency to correlate with CSF NFL (Rho = 0.289; *P* = 0.094, respectively). Finally, psychosocial MFIS correlated with 9-HPT (Rho = 0.303; *P* = 0.021). The BDI-II scores did not correlate with any of the clinical variables or biomarkers evaluated.

PROs measures were not different regarding gender, disease activity during study time, presence of intrathecal synthesis of immunoglobulin M (IgM oligoclonal bands), need of treatment escalation, or season of the year at time of completion of the questionnaire. However, patients with lower baseline MFIS scores (global and all subscales) were on first-line DMT and those with worse measures were on high-efficacy DMT (*P* = 0.008). This difference was not observed for MusiQoL (*P* = 0.074) or BDI-II (*P* = 0.199).

### Prognostic value of baseline PROs after two-year follow-up in pwRMS cohort

The median change in EDSS in the cohort of pwRMS was 0 [− 0.375 to 0.5] at the end of the study. Two-year EDSS change correlated with global MFIS (Rho = 0.427; *P* = 0.007), subscales physical (Rho = 0.453; *P* = 0.004), cognitive (Rho = 0.333; *P* = 0.038), and psychosocial (Rho = 0.511; *P* = 0.001), also with BDI-II (Rho = 0.531; *P* = 0.002), but not with MusiQoL (Rho = − 0.272; *P* = 0.125) (Table 3).

**Figure 2:**
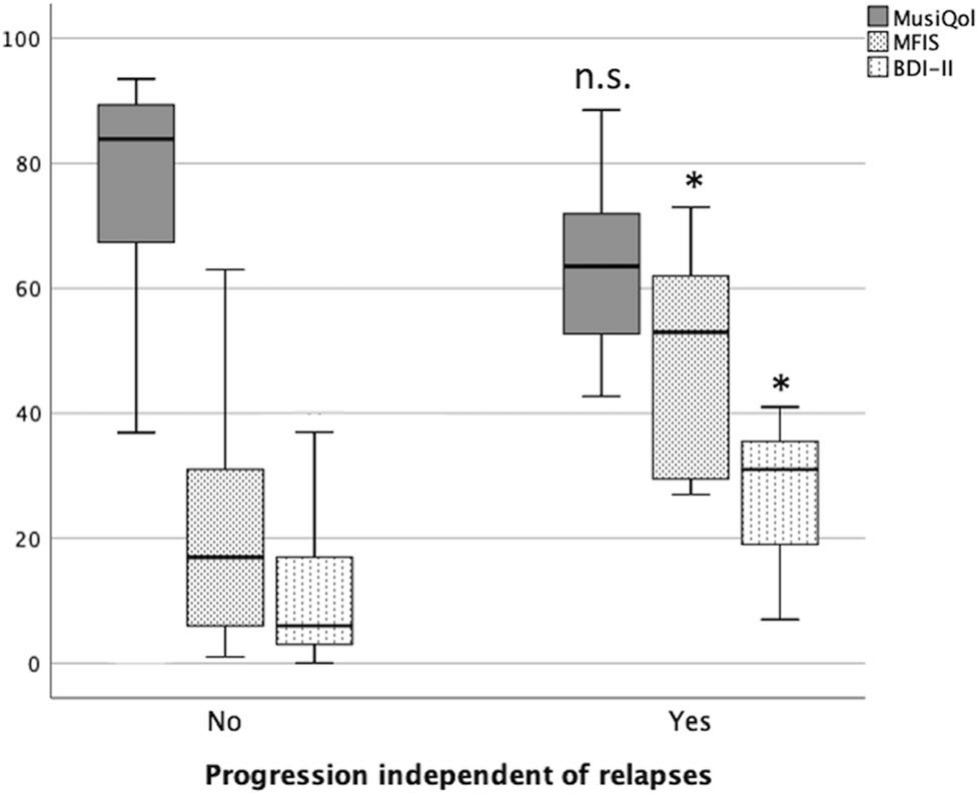
Association between progression independent of relapse (PIRA) and baseline PROs results. Box plot showing PRO results in patients that experienced progression-independent of relapse disability (PIRA) compared to those that did not at the end of the study period (2-year). Although all scales worsened in patients with PIRA, MFIS and BDI-II scales were significantly higher in these patients. Abbreviations: PRO: patient-reported outcomes; MusiQoL: Multiple Sclerosis Quality of Life; MFIS: Modified Fatigue Impact Scale; BDI-II: Beck Depression Inventory-II; PIRA: progression-independent of relapse disability.

Seventeen pwRMS experienced DW at the end of the observation period. Although 36 patients relapsed, DW associated with disease activity (RAW) occurred only in 4 of them. Fifteen patients accomplished criteria for DW independent of relapse (PIRA). PIRA was more frequent in pwRMS with higher scores in MFIS after adjustment for age, disease duration, DMT, EDSS, and BDI-II scores. In the bivariate analysis, baseline EDSS (*P* = 0.006) and BDI-II (*P* = 0.000) were extracted as influencing covariates, but the multivariate analysis confirmed MFIS as an independent predictor of PIRA (*P* = 0.009). Higher values in BDI-II were also more frequent in pwRMS with PIRA (*P* = 0.002) also adjusting for EDSS and MFIS, while MusiQoL did not predict PIRA (*P* = 0.592) (Fig. 2).

### Suggested global MFIS cut-off values for detection and prediction of progression

**Figure 3:**
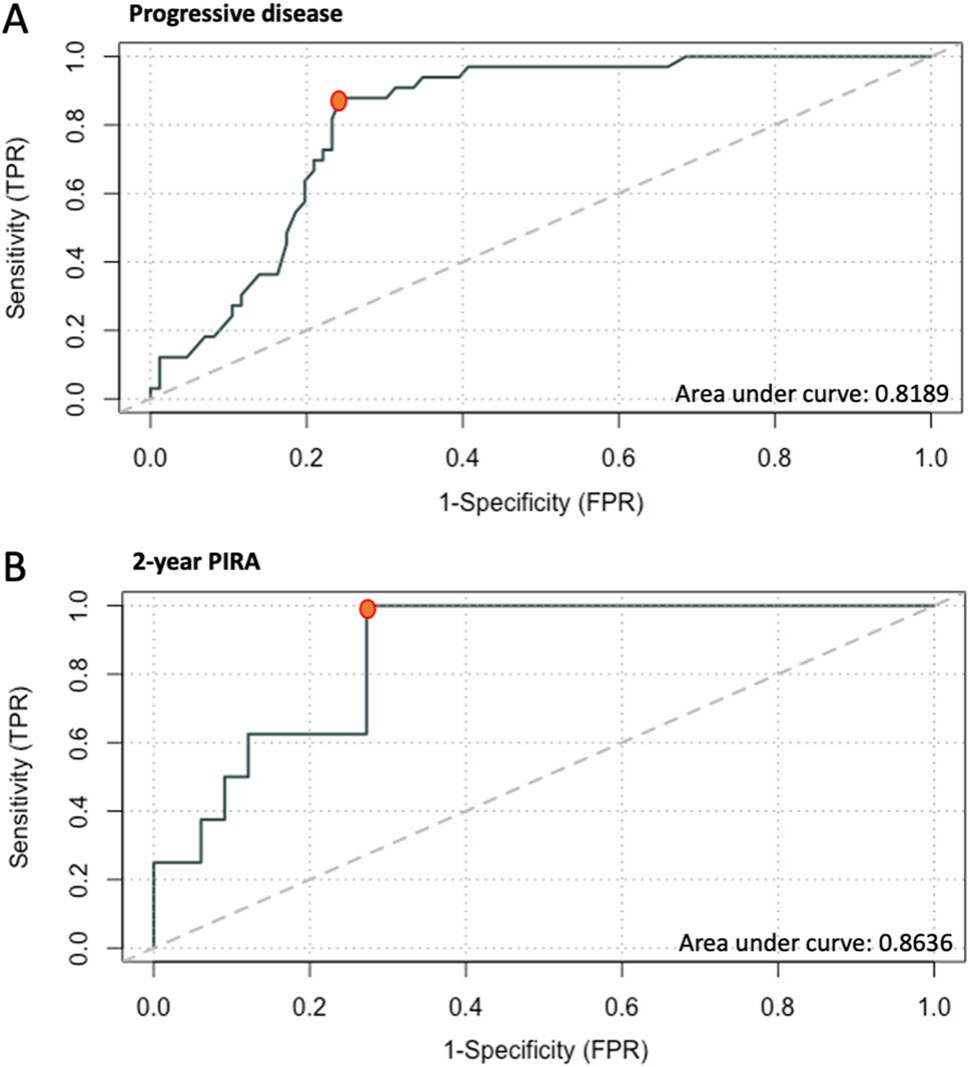
Receiver operating characteristics (ROC) curve analysis to obtain global MFIS cut-off values for prediction of progressive disease. (**A**) ROC curve to calculate a cut-off value by comparing global MFIS score between pwPMS and pwRMS cohorts (**B**) ROC curve to calculate a cut-off value by comparing global MFIS score between patients with and/or without PIRA in the pwRMS cohort. Abbreviations: pwPMS: person with progressive MS; pwRMS: person with relapsing MS; MFIS: Modified Fatigue Impact Scale;PIRA: progression-independent of relapse disability.

We aimed to determine cut-off values for classification of pwRMS in function of results from MFIS questionnaires. First, receiver operating characteristic curve (ROC) was used to calculate a cut-off value by comparing global MFIS score between pwPMS and pwRMS cohorts (Fig. 3). ROC analysis resulted in a most optimal cut-off point of 39.5 (specificity of 76.7%; sensitivity 81.8%; accuracy 78.1%) that discriminated significantly between pwPMS and pwRMS (Chi-square *P* < 0.000). This cut-off point, when applied to the prospective pwRMS cohort, resulted in a specificity of 87,9%; sensitivity of 62.5%; accuracy of 82.9% to distinguish between pwRMS with or without PIRA (Chi-square *P* = 0.022). Secondly, an alternative cut-off point was calculated among pwRMS in the prospective cohort comparing those that experienced PIRA after 2-years with those that did not. The most optimal cut-off score extracted was 28, which resulted in a specificity of 72.7%; sensitivity of 87.5%; accuracy of 75.6% (Chi-square *P* = 0.003).

## Discussion

This study shows that the PRO questionnaires distinguished well between pwRMS and pwPMS, and that within a prospective cohort of pwRMS there was heterogeneity in the results retrieved from them. In pwRMS a significant correlation between baseline PRO and disability measures was found. Global MFIS, and particularly physical MFIS, were the most informative PRO regarding current neurological disability. Furthermore, two-year EDSS change and PIRA were associated to higher global MFIS and BDI-II scores suggesting a potential usefulness of baseline PRO measures to detect patients at risk of progression.

PRO questionnaires are increasingly being used in MS clinical practice with the aim of evolving towards a patient-centered management of the disease ^5^. Although the methodology still lacks established standards, the benefits of its integration into clinical routine cannot be ignored. A wide range of PRO with different purposes have been applied in MS so far, however only few PRO give adequate psychometric quality^5^. In this regard, MusiQoL, MFIS, and BDI-II are standardized PRO questionnaires that are suitable to evaluate QoL, fatigue, and depression, respectively, in MS patients^7–9^. In our work, all PRO investigated correlated between each other within both pwPMS and pwRMS cohorts. In line with our results, it has been shown that MFIS and BDI-II correlate between them and with QoL and health status^10^. Accordingly, we showed that the two cohorts pwRMS and pwPMS had significantly different scores in the investigated PRO. Although not all studies found this association^11^, several publications indicate a potential role for PRO as suitable indicators of the disease pattern of MS patients^10,12–16^.

Robust correlations between health status and disability level have been reported in the literature^17^. MusiQol, MFIS, and BDI-II are correlated with neurological disability in pwRMS^10,12^. In our cohort, EDSS correlated with all questionnaires, although when correcting for multiple comparisons, only correlations with MFIS and its subdomains remained significant. With regard to other clinical parameters of disability, global MFIS, and particularly, the physical MFIS domain, were correlated with 9-HPT and showed a trend to correlate with T25-FW and SDMT. Similarly, it has been previously reported how physical and cognitive fatigue could be associated with visual dysfunction, cognition, and neurologic impairment^18^.

Among several MS biomarkers, CSF chitinase 3–like 1 (CHI3L1) has been linked to the ongoing neuroinflammatory process and reactive gliosis that occur in advanced disease^19^. NFL, in the CSF or serum, a biomarker for acute axonal injury, has been mainly proposed as surrogate for disease activity in MS^20^, and potentially, it has been related to prospective neurological disability and cerebral atrophy^21^. A report by our group showed that the combined measure of CSF CHI3L1 and NFL could be useful to discriminate MS phenotypes and that higher levels of both biomarkers might predict clinical progression^22^. For these reasons, CSF CHI3L1 and NFL, and baseline serum NFL were investigated. MusiQoL scores correlated with CSF and serum NFL, although association with other clinical parameters was not significant. A feasible explanation for the correlation with high CSF NFL levels could be that disease activity when patients underwent the lumbar puncture resulted in permanent sequelae, and consequently, in a loss of QoL. Correlation with elevated serum NFL could be explained by contemporary disease activity. The analysis of the MFIS cognitive domain alone, unmasked the correlation between fatigue and levels of CSF CHI3L1. High levels of CHI3L1 have been associated with neurologic disability explained by its expression in chronic active lesions in pwPMS, not only in white matter but also diffusely in the cortex, in close relationship to degenerating axons^23^. Thus, both the physical and cognitive MFIS domains, seemed to be complementary in describing disability in pwRMS, and the potential differences may provide insight into the underlying pathogenic mechanisms of fatigue^18^. A study by Hakansson et al., did not find correlation between fatigue scores, clinical and biomarker parameters in a population of 38 CIS and RMS patients^24^. The discrepancy between our study and theirs might be related to a lower sample size that precluded them for obtaining statistical significance, and/or to the presence of CIS patients in the study cohort in which, disability was low, and if existent, the underlying mechanisms of progression that determine an increase in CSF biomarkers of advanced disease, such as CHI3L1, might also be underrepresented.

Finally, we investigated the value of baseline PRO to predict EDSS worsening and whether this change in disability was dependent or independent on relapses. MusiQoL did not predict PIRA or RAW in our cohort. Although several studies have found a relationship between MusiQoL and long-term disability^25,26^, other found that only specific dimensions (sentimental and sexual) were associated with responsiveness to EDSS change^27^. In contrast, we found that the BDI-II and global MFIS scores could predict PIRA, but not RAW, after a 2-year follow-up. Depression is a strong determinant of poor QoL in patients with MS^10^, shares several features with fatigue , and both have a similar longitudinal course^28,29^. Despite the correlation between depression and fatigue, BDI-II did not reflect baseline disability, and although it was predictive of disability progression, it was not an influencing covariate on the predictive role of MFIS in PIRA. Thus, although there might be either common underlying mechanisms or interdependence by a cause-and-effect relationship between fatigue and depression^29–32^, there are differential aspects that may account for the predominant association of MFIS with clinical disability.

The results of the Comprehensive Longitudinal Investigation of Multiple Sclerosis at Brigham and Women’s Hospital (CLIMB) indicated that patients in the period closely preceding the transition from RMS to PMS had worse physical QoL and fatigue compared to subjects who remained RMS^33^. Baseline fatigue was also predictive of EDSS worsening in a retrospective cohort of pwMS of the New York State MS Consortium, although the study design did not allow to specifically assess and adjust the effect of depression^34^. Other retrospective studies have also reported similar results^11^. Prospective data published from the clinical trial ORATORIO, in a larger cohort of randomized and controlled primary progressive MS patients, showed that those experiencing PIRA had a significantly greater increase in fatigue (in all three MFIS subscales) than patients who did not^35^. Due to the potential value of the baseline MFIS measure to detect and/or predict disability, our objective was to establish a reference value with a clinical purpose . Generally, studies use a total score of 38 as a cut-off point to discriminate fatigued from non-fatigued individuals^13,36^ based on a study that correlated MFIS with another fatigue inventory^30^. This cut-off point has been questioned in other reports^37^. From our analysis, a value of 39.5 in global MFIS allowed us to distinguish between pwPMS and pwRMS with a good specificity and sensitivity profile, similar to what had been described for fatigued and non-fatigued patients. However, for pwRMS that experienced PIRA, this cut-off point was less sensitive. An alternative ROC analysis in pwRMS regarding whether PIRA occurred or not, suggested a more optimal cut-off value of 28 that could detect earlier in time those patients at risk of progression.

Our study has several limitations. Firstly, the cohort of pwPMS was retrospective and, secondly, PIRA and RAW events were low during the 2-year study period. Also, from the prospective cohort of pwRMS that entered the study, only 45–66% of patients participated actively in completing the scales. As a real-world study, patients were invited to complete the questionnaires, but not all were sufficiently involved, and we could not retrieve their inputs. This could cause a potential response bias, as more affected patients might be more prone to complete the forms. Similarly, a large-scale longitudinal study involving 2356 participants retrieved PRO measures only in 808 patients (34.3%)^33^. This confirms that, although PRO might be a useful tool in clinical practice, more efforts are needed to convince patients, physicians, and health-related professionals about the worthiness to integrate the patient’s opinion in the daily practice equation.

In conclusion, in this report we show that the information gathered from people with MS provides useful information about their current status, as a snapshot, but also might predict the course of the disease. Thus, we consider that together with other clinical variables and biomarkers, global MFIS and its domains constitute a diagnostic and prognostic aid in clinical practice for the design of personalized therapeutic strategies.

## Material and methods

### Participants and study design

**Figure 4:**
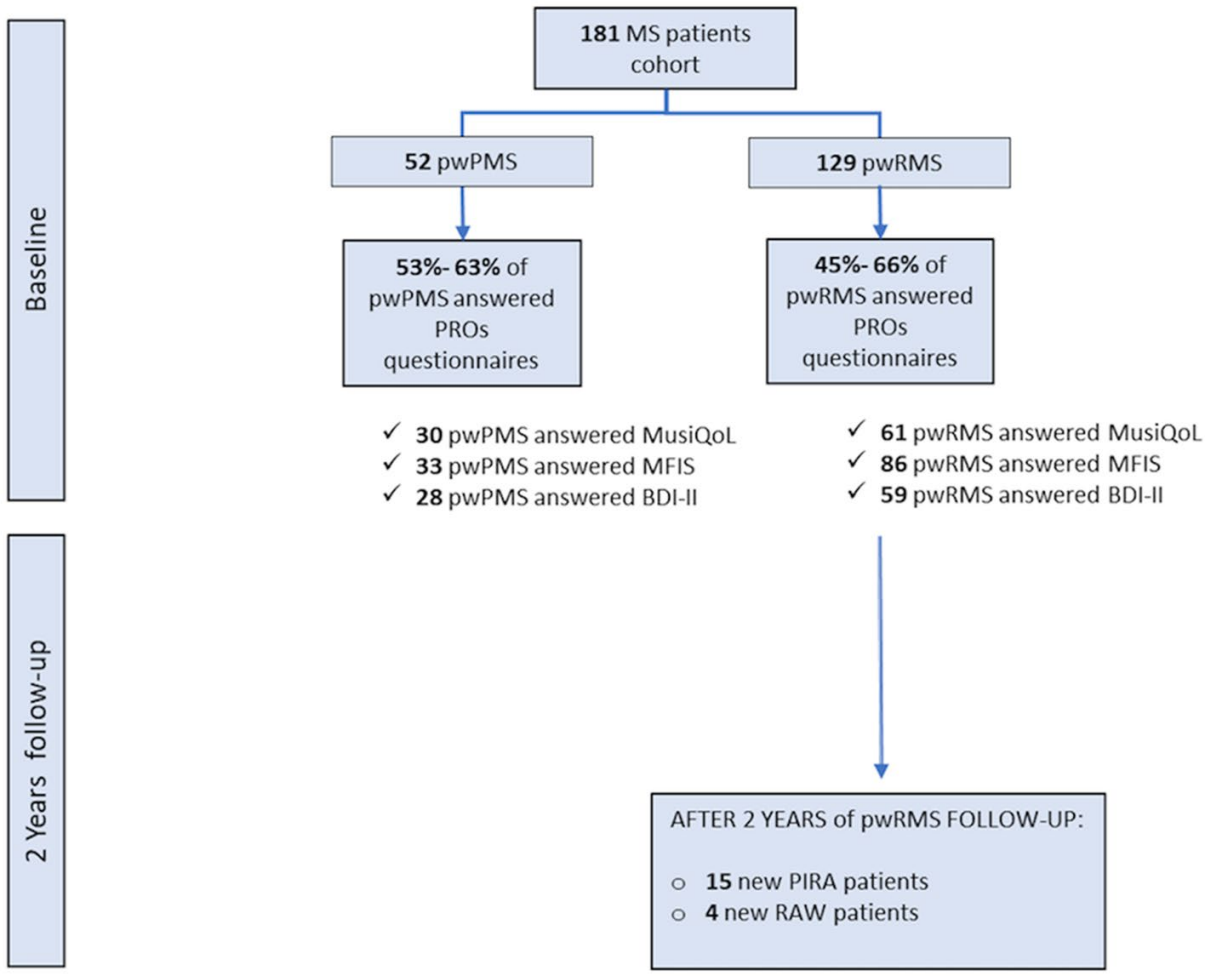
Flow chart of study cohort. Two distinct groups of patients were selected at study inclusion, a retrospective cohort of pwPMS and a prospective cohort of pwRMS. From the patients that entered the study, there was a loss of patients that did not fill in the questionnaires. Patients were followed for 2 years, and baseline PRO measures, clinical and biochemical variables were included in the database. During the 2-year study period, the presence of disease activity (relapse and/or gadolinium-enhancing lesions/new T2 lesions in MRI) was registered, as well as disability change due to disease activity (RAW) and/or to progression independent of relapse (PIRA). Abbreviations: MS: multiple sclerosis; pwPMS: person with progressive MS; pwRMS: person with relapsing MS; PRO: patient-reported outcomes; MusiQoL: Multiple Sclerosis Quality of Life; MFIS: Modified Fatigue Impact Scale; BDI-II: Beck Depression Inventory-II; PIRA: progression-independent of relapse disability; RAW: relapse-associated worsening.

This is a real-world observational study in a single center – the University and Polytechnic Hospital La Fe, Valencia- designed to perform the following analyses:

- Cross-sectional description of PRO in two cohorts: pwRMS (n = 86) (prospective) and persons with PMS (pwPMS) (n = 30) (retrospective). These patients constitute a percentage from two initial larger cohorts selected if they filled in PRO questionnaires at study initiation (Fig. 4). We compared baseline demographics and clinical variables between both populations consisting of those who completed the questionnaire and those who did not, but we found no significant difference, including cognitive function (SDMT) or global disability (EDSS).
- Baseline PRO characterization of the prospective cohort of pwRMS and association of results with distinctive clinical and biochemical variables.
- Investigation of the potential prediction of clinical outcomes by PRO—presence of disease activity, EDSS change, relapse-associated disability worsening (RAW), and progression independent of relapses (PIRA)- after a 2-year follow-up.

Patients were recruited in the period that included between October 2019 and July 2020 and the inclusion criteria were as follows:

- pwPMS: patients with progressive disease according to McDonald criteria^3,38^ that were capable to fill up the PRO questionnaires.
- pwRMS: patients under 40 years of age, <10 years of MS diagnosis, absence of progressive disease at inclusion time, Expanded Disability Status Scale (EDSS) < 3.5^39^.

All research was carried out according to with relevant local regulations. Patients were informed about the study, they accepted to participate, and their written consent was obtained. The study was approved by the Institutional Ethics Committee in the Hospital Universitari Ia Politècnic La Fe.

### Variables studied

For pwPMS, the following variables were registered at baseline: date of birth, date of MS diagnosis, sex, baseline age and disease duration, current disease-modifying therapy (DMT), PRO measures, and date of completion.

For pwRMS, the same variables were recorded at baseline plus cerebrospinal fluid (CSF) neurofilament light chain protein (NFL) and chitinase3 like-1 (CHI3L1) levels, and CSF oligoclonal G and M bands (for Methods^22^). CSF biomarkers were available in 50 pwRMS (58.1%). During the 2-year study period and every three to six months, serum NFL – measured with SiMoA SR-X according to manufacturer’s instructions [Quanterix Corporation, Lexington, MA 02,421, USA]—EDSS, symbol digit modality test (SDMT)^40^, 9-hole peg test (9-HPT)^41^, and timed 25-foot walk test (T25-FW) were registered^41^.

### Clinical definitions

MS active disease was defined as a clinical attack and/or, at least, one gadolinium enhanced lesion (GEL) in T1-weighted MRI, and/or a new lesion in T2-weighted MRI. Relapse was defined as acute worsening of neurologic function occurred lasting more than 24 h, not explained by fever or physical stress. The disability worsening (DW) was defined as an increase in EDSS (≥ 1.0 points if the baseline EDSS was lower than 5.5) or ≥ 0.5 points if the baseline EDSS was ≥ 5.5 points) that was confirmed after 3/6 months of the initial assessment. DW events were ultimately classified as relapse-associated worsening (RAW) if they took place within 90 days from the onset of a reported relapse or as progression independent of relapse activity (PIRA) if a confirmed DW event took place in the absence of a reported relapse within the last 6 months^42^.

### Patient-reported outcomes (PRO)

Patient self-administration questionnaires were contemporaneous to the registration of baseline clinical variables and biomarker analysis. Three validated standardized PRO questionnaires in their Spanish version were evaluated: MusiQoL^43^, Modified Fatigue Impact Scale (MFIS)^44^ and Beck Depression Inventory II (BDI-II)^45^. For a further detailed description of questionnaires and domains, see Supplementary Methods (Annex 1). After a detailed explanation of the questionnaires, most of the patients completed a pseudonymized online form for clinical purposes outside the outpatient clinic, with the exemption of those who lacked internet devices or web navigation skills that filled up a paper form after the clinical visit. Those in the PMS cohort whose disability limited their writing ability were assisted by their caregiver.

### Treatments

pwRMS were treated with DMT according to routine clinical practice. In general, a platform or oral first line DMT (fl-DMT) was offered unless any of the following circumstances occurred: (i) two clinical attacks in a year, (ii) a clinical attack and/or a new gadolinium-enhanced lesion (GEL) within a year, or (iii) a disabling clinical attack with residual EDSS of at least 2 points in the pyramidal system. In these cases, and in those with treatment failure, high efficacy DMT (he-DMT) were preferred. We considered as he-DMT: cladribine, fingolimod, natalizumab, anti-CD20 monoclonal antibodies, alemtuzumab, and haematopoietic stem cell transplantation (aHSCT).

### Statistical analysis

Continuous variables were reported as mean (SD) or median, p25–p75, and categoric variables as absolute frequencies and percentages. Data did not distribute normally and were analyzed with nonparametric tests. Groups were compared with Mann–Whitney U test and correlations with Spearman’s test. Two-sided *p*-value less than 0.05 was considered to indicate statistically significant differences. Multiple comparisons were adjusted with Benjamini–Hochberg procedure to decrease false discovery rate (FDR). General lineal model-univariate and multivariate analyses of variance were performed with sex, baseline age and disease duration, DMT, EDSS, and BDI-II as covariates. Season of the year was also used as covariate for MFIS associations. Receiver-operating (ROC) curve analyses were performed to obtain cut-off values for classification of pwMS regarding progressive disease and/or PIRA. Statistical analyses were performed using R studio 2021.09 (https://www.R-project.org/”).

## Supplementary Information

Supplementary Information.

## Data Availability

The datasets generated during and/or analysed during the current study are available from the corresponding author on reasonable request.

## References

[1] Oreja-Guevara C (2020). PND22 discover study, first analysis specific for secondary progressive multiple sclerosis burden and cost in Spain: interim analysis results. Value Health.

[2] Confavreux C, Vukusic S (2006). Natural history of multiple sclerosis: a unifying concept. Brain.

[3] Thompson AJ (2018). Diagnosis of multiple sclerosis: 2017 revisions of the McDonald criteria. Lancet Neurol..

[4] Krajnc N, Bsteh G, Berger T (2021). Clinical and paraclinical biomarkers and the hitches to assess conversion to secondary progressive multiple sclerosis: a systematic review. Front. Neurol..

[5] D'Amico E, Haase R, Ziemssen T (2019). Review: patient-reported outcomes in multiple sclerosis care. Mult. Scler. Relat. Disord..

[6] Filippi, M. *et al.* Multiple sclerosis. *Nat Rev Dis Primers* **4**, (2018). 10.1038/s41572-018-0041-4 30410033

[7] Fernández O (2011). Validation of the spanish version of the multiple sclerosis international quality of life (musiqol) questionnaire. BMC Neurol..

[8] Sanz J, García-Vera MP (2013). Diagnostic performance and factorial structure of the beck depression inventory-second edition (BDI-II). Anal. Psicol..

[9] Kos D (2005). Evaluation of the Modified fatigue impact scale in four different European countries. Mult. Scler..

[10] Reese JP (2013). Preference-based Health status in a German outpatient cohort with multiple sclerosis. Health Qual Life Outcomes.

[11] Ghajarzadeh M (2013). Fatigue in multiple sclerosis: Relationship with disease duration, physical disability, disease pattern, age and sex. Acta Neurol. Belg..

[12] Farran N (2020). Factors affecting MS patients’ health-related quality of life and measurement challenges in Lebanon and the MENA region. Mult. Scler. J. Exp. Transl. Clin..

[13] Kos D (2005). Evaluation of the modified fatigue impact scale in four different European countries. Mult. Scler. J..

[14] Marchesi O (2020). Fatigue in multiple sclerosis patients with different clinical phenotypes: a clinical and magnetic resonance imaging study. Eur. J. Neurol..

[15] Zhang Y, Taylor B, van der Mei I (2017). Patient-reported outcomes are worse for progressive-onset MS than relapse-onset MS, particularly early in the disease process. Mult. Scler. J..

[16] Rooney S, Wood L, Moffat F, Paul L (2019). Prevalence of fatigue and its association with clinical features in progressive and non-progressive forms of multiple sclerosis. Mult. Scler. Relat. Disord..

[17] Mitchell AJ, Benito-Leon J, Gonzalez JM, Rivera-Navarro J (2005). Quality of life and its assessment in multiple sclerosis: integrating physical and psychological components of wellbeing. Lancet Neurol..

[18] Chahin S (2015). Relation of quantitative visual and neurologic outcomes to fatigue in multiple sclerosis. Mult. Scler. Relat. Disord..

[19] Floro S (2022). Role of Chitinase 3–like 1 as a biomarker in multiple sclerosis. Neurol. Neuroimmunol. Neuroinflammation.

[20] Khalil M (2018). Neurofilaments as biomarkers in neurological disorders. Nat. Rev. Neurol..

[21] Kuhle J (2017). Serum neurofilament is associated with progression of brain atrophy and disability in early MS. Neurology.

[22] Gil-Perotin S (2019). Combined cerebrospinal fluid neurofilament light chain protein and Chitinase-3 like-1 levels in defining disease course and prognosis in multiple sclerosis. Front Neurol.

[23] Cubas-Núñez, L. *et al.* Potential role of CHI3L1+ astrocytes in progression in MS. *Neurol. Neuroimmunol. Neuroinflammation.* **8**, (2021). 10.1212/NXI.0000000000000972 PMC7931642 33658322

[24] Håkansson I, Johansson L, Dahle C, Vrethem M, Ernerudh J (2019). Fatigue scores correlate with other self-assessment data, but not with clinical and biomarker parameters, in CIS and RRMS. Mult. Scler. Relat. Disord..

[25] Al Jumah M (2021). A prospective multicenter study for assessing MusiQoL validity among Arabic-speaking MS patients treated with subcutaneous interferon β-1a. Mult. Scler. Int..

[26] Baumstarck K (2013). Health-related quality of life as an independent predictor of long-term disability for patients with relapsing–remitting multiple sclerosis. Eur. J. Neurol..

[27] Baumstarck K (2013). Responsiveness of the multiple sclerosis international quality of life questionnaire to disability change: a longitudinal study. Health Qual Life Outcomes.

[28] Greeke EE (2017). Depression and fatigue in patients with multiple sclerosis. J. Neurol. Sci..

[29] Bakshi R (2000). Fatigue in multiple sclerosis and its relationship to depression and neurologic disability. Mult. Scler..

[30] Flachenecker P (2022). Fatigue in multiple sclerosis: a comparison of different rating scales and correlation to clinical parameters. Mult. Scler..

[31] Gobbi C (2014). Forceps minor damage and co-occurrence of depression and fatigue in multiple sclerosis. Mult. Scler. J..

[32] Palotai M (2021). Microstructural changes in the left mesocorticolimbic pathway are associated with the comorbid development of fatigue and depression in multiple sclerosis. J. Neuroimag..

[33] Healy BC, Zurawski J, Chitnis T, Weiner HL, Glanz BI (2021). Patient-reported outcomes associated with transition to secondary progressive multiple sclerosis. Qual. Life Res..

[34] Vaughn CB (2020). Fatigue at enrollment predicts EDSS worsening in the New York state multiple sclerosis consortium. Mult. Scler. J..

[35] Miller, D. *et al.* The association between confirmed disability progression and patient-reported fatigue in PPMS patients in the ORATORIO study (S33.006). *Neurology* **88**, (2017).

[36] Téllez N (2005). Does the modified fatigue impact scale offer a more comprehensive assessment of fatigue in MS?. Mult. Scler..

[37] Larson RD (2013). Psychometric properties of the modified fatigue impact scale. Int. J. MS Care.

[38] Polman CH (2011). Diagnostic criteria for multiple sclerosis: 2010 revisions to the McDonald criteria. Ann. Neurol..

[39] Kurtzke JF (1983). Rating neurologic impairment in multiple sclerosis: an expanded disability status scale (EDSS). Neurology.

[40] Sumowski JF (2018). Cognition in multiple sclerosis: state of the field and priorities for the future. Neurology.

[41] Kragt JJ, van der Linden FAH, Nielsen JM, Uitdehaag BMJ, Polman CH (2006). Clinical impact of 20% worsening on timed 25-foot Walk and 9-hole peg test in multiple sclerosis. Mult. Scler. J..

[42] Kappos L (2020). Contribution of relapse-independent progression versus relapse-associated worsening to overall confirmed disability accumulation in typical relapsing multiple sclerosis in a pooled analysis of 2 randomized clinical trials. JAMA Neurol..

[43] Fernández O (2011). Validation of the spanish version of the multiple sclerosis international quality of life (musiqol) questionnaire. BMC Neurol..

[44] Kos D (2005). Evaluation of the modified fatigue impact scale in four different European countries. Mult. Scler..

[45] Sanz J, Perdigón AL, Vázquez C (2003). Adaptación española del inventario para la Depresión de Beck-II (BDI-II): 2. Propiedades psicométricas en población general. Clin. Salud..

