# Supplementary material for: Patient's perspective in clinical practice to assess and predict disability in multiple sclerosis
Source: Sci Rep. 2022 Oct 29;12:18238. doi: 10.1038/s41598-022-23088-x (PMC9617913; doi:10.1038/s41598-022-23088-x)
Supplement: Supplementary file 1 — Supplementary Information. [file 41598_2022_23088_MOESM1_ESM.docx]

**Supplementary Methods Annex 1. Description of patient-reported questionnaires (PROs)**

MusiQoL (1,2), is a MS-specific scale that represents health status in pwMS within the 4 last weeks. It comprises 31 items that describe nine dimensions named according to its constitutive items: activities of daily living psychological well-being, symptoms, relationships with friends, relationships with family, relationship with the healthcare system, sentimental and sexual life, coping, and. Each item was answered using a six-point Likert scale. All dimension scores were linearly transformed to a 0-100 scale where 0 represents the worst possible QoL and 100 the best possible level of QoL.

The MFIS scale is a 21-item MS-specific scale that assesses within the 4 last weeks the impact of fatigue in three function domains: cognitive, physical, and psychosocial (3, 4). The total score for the MFIS is the sum of the scores for the 21 items in each of the three domains or subscales. The score ranges from 0 to 84, with higher scores indicating greater fatigue. In the literature, a cut-off value of 38 has been used to distinguish fatigued from non-fatigued patients (5).

Depression Inventory II (BDI-II) is one of the most widely used psychometric test for measuring the severity of depression (6). It is not MS-specific and refers to the 2 last weeks. It consists of a 21-item, with each answer scored on a scale value of 0 to 3. Higher total scores indicate more severe depressive symptoms. The standardized *cut-offs* are: 1) 0–13: minimal depression, 2) 14–19: mild depression, 3) 20–28: moderate depression and 4) 29–63: severe depression.

1. Simeoni M, Auquier P, Fernandez O et al (2008) MusiQol study group. Validation of the Multiple Sclerosis International Quality of Life Questionnaire. Mult Scler 14(2):219–230
2. Mitchell AJ, Benito-Leon J, Gonzalez JM, Rivera-Navarro J. Quality of life and its assessment in multiple sclerosis: integrating physical and psychological components of wellbeing. Lancet Neurol. 2005;4:556–66.
3. Fisk JD, Ritvo PG, Ross L, Haase DA, Marrie TJ, Schlech WF. Measuring the functional impact of fatigue: initial validation of the fatigue impact scale. Clin Infect Dis. 1994 Jan;18 Suppl 1:S79-83. doi: 10.1093/clinids/18.supplement_1.s79. PMID: 8148458.
4. Kos D., Kerckhofs E., Carrea I., Verza R., Ramos M., Jansa J. Evaluation of the Modified Fatigue Impact Scale in four different European countries. Mult. Scler. J. 2005;11:76–80. doi: 10.1191/1352458505ms1117oa.
5. Flachenecker P, Kumpfel T, Kallmann B, Gottschalk M, Grauer O, Rieckmann P, et al. Fatigue in multiple sclerosis: a comparison of different rating scales and correlation to clinical parameters. Mult Scler. 2022;8:523–6.
6. Beck, A. T., Steer, R. A., & Brown, G. K. (1996b). Manual for Beck Depression Inventory-II. San Antonio, TX. Psychological Corporation
